# In Vivo Toxicity Evaluation of Sugar Adulterated *Heterotrigona itama* Honey Using Zebrafish Model

**DOI:** 10.3390/molecules26206222

**Published:** 2021-10-15

**Authors:** Rafieh Fakhlaei, Jinap Selamat, Ahmad Faizal Abdull Razis, Rashidah Sukor, Syahida Ahmad, Arman Amani Babadi, Alfi Khatib

**Affiliations:** 1 Food Safety and Food Integrity (FOSFI), Institute of Tropical Agriculture and Food Security, Universiti Putra Malaysia, Serdang 43400, Selangor, Malaysia; rafieh.fakhlaei@gmail.com (R.F.); rashidah@upm.edu.my (R.S.); 2 Department of Food Science, Faculty of Food Science and Technology, Universiti Putra Malaysia, Serdang 43400, Selangor, Malaysia; madfaizal@upm.edu.my; 3 Natural Medicines and Products Research Laboratory, Institute of Bioscience, Universiti Putra Malaysia, Serdang 43400, Selangor, Malaysia; 4 Department of Biochemistry, Faculty of Biotechnology & Biomolecular Sciences, Universiti Putra Malaysia, Serdang 43400, Selangor, Malaysia; syahida@upm.edu.my; 5 Department of Molecular Medicine, School of Advanced Technologies in Medicine, Tehran University of Medical Sciences, Tehran 55469-14177, Iran; ar.amani65@gmail.com; 6 Department of Pharmaceutical Chemistry, Kulliyyah of Pharmacy, International Islamic University Malaysia, Kuantan 25200, Pahang, Malaysia; alfikhatib@iium.edu.my

**Keywords:** *Heterotigona itama* honey, honey quality, adulteration, *Danio rerio*, toxicity assessment

## Abstract

Honey is prone to be adulterated through mixing with sugars, cheap and low-quality honey, and other adulterants. Consumption of adulterated honey may cause several health issues such as weight gain, diabetes, and liver and kidney dysfunction. Therefore, studying the impact of consumption of adulterated honey on consumers is critical since there is a lack of study in this field. Hence, the aims of this paper were: (1) to determine the lethal concentration (LC_50_) of adulterated honey using zebrafish embryo, (2) to elucidate toxicology of selected adulterated honey based on lethal dose (LD_50_) using adult zebrafish, (3) to determine the effects of adulterated honey on histological changes of zebrafish, and (4) to screen the metabolites profile of adulterated honey by using zebrafish blood serum. The LC_50_ of *Heterotrigona itama* honey (acacia honey) and its sugar adulterants (light corn sugar, cane sugar, inverted sugar, and palm sugar in the proportion of 1–3% (*w*/*w*) from the total volume) was determined by the toxicological assessment of honey samples on zebrafish embryos (different exposure concentrations in 24, 48, 72, and 96 h postfertilization (hpf)). Pure *H. itama* honey represents the LC_50_ of 34.40 ± 1.84 (mg/mL) at 96 hpf, while the inverted sugar represents the lowest LC_50_ (5.03 ± 0.92 mg/mL) among sugar adulterants. The highest concentration (3%) of sugar adulterants were used to study the toxicology of adulterated honey using adult zebrafish in terms of acute, prolong-acute, and sub-acute tests. The results of the LD_50_ from the sub-acute toxicity test of pure *H. itama* honey was 2.33 ± 0.24 (mg/mL). The histological studies of internal organs showed a lesion in the liver, kidney, and spleen of adulterated treated-honey groups compared to the control group. Furthermore, the LC-MS/MS results revealed three endogenous metabolites in both the pure and adulterated honey treated groups, as follows: (1) S-Cysteinosuccinic acid, (2) 2,3-Diphosphoglyceric acid, and (3) Cysteinyl-Tyrosine. The results of this study demonstrated that adulterated honey caused mortality, which contributes to higher toxicity, and also suggested that the zebrafish toxicity test could be a standard method for assessing the potential toxicity of other hazardous food additives. The information gained from this research will permit an evaluation of the potential risk associated with the consumption of adulterated compared to pure honey.

## 1. Introduction

The standards of *Codex Alimentarius* [1] define honey as the natural sweet substance produced by honey bees from the nectar of plants or secretions of living parts of plants. The honeybees can collect and transform it by mixing it with specific substances of their own, then deposit it, dehydrate it, and store it in the honeycomb to ripen and mature. The harvested honey from honeybees can be consumed not only as a sweetener but also as a medicine due to its therapeutic effect on human health.

Honey contains mainly sugar and more than 200 other compounds [2]. Sugars, as a major component of carbohydrates, comprise 95–99% of honey’s dry matter [3]. Fructose and glucose are the main sugars in *H. itama* honey in the proportion of 13.00 ± 0.21 (g/100 g honey) for the former and 11.69 ± 0.56 (g/100 g honey) for the latter [4]. In addition to fructose and glucose, several other disaccharides and oligosaccharides, including sucrose 0.31 ± 0.01 (g/100 g honey), maltose 22.56 ± 1.22 (g/100 g honey), maltotriose, and panose, can be found. Organic acids, minerals, and trace elements such as calcium, potassium, sodium, magnesium, phosphorus, sulphur, iron, zinc, copper, and manganese are other components presented in pure *H. itama* [3].

In general, honey is perceived as a high-quality product and the most susceptible to be adulterated or have incorrect labelling and fraudulent mixing with lower-cost and low-quality honey, sugars, and other substances. Consequently, owing to therapeutic properties, limited availability, and high prices, honey is subjected to adulteration. Adulterants are referring to any substances that are added to the original and pure product to corrupt, debase, or prepare for sale by replacing more valuable substances with less valuable ones. Honey can be adulterated directly (bee-feeding) or indirectly (addition of adulterants). Cheap sweeteners such as cane sugar, corn syrup, maltose, and high fructose syrup are being used commonly to adulterate honey [5]. Honey adulteration has a major impact on economic loss not only due to declining honey quality but also by making difficulties in the marketing of pure honey [6]. Honey has been targeted for adulteration worldwide due to the high demand [7]. Therefore, international committees have focused on the quality control of honey and safety protocols.

Inexpensive sugars or industrial syrups are generally used for the adulteration of honey. Soares, et al. [8] described well-known adulterants produced from sugar cane or sugar beet, such as sugar syrups, corn syrup (CS) and high-fructose corn syrup (HFCS), glucose syrup (GS), sucrose syrup, inverted syrup (IS), or high-fructose inulin syrup (HFIS). These sugars may replicate the natural taste of the honey while increasing the yield volume, but the lack of nutrition and therapeutic substances are unavoidable. Moreover, the addition of these sugars may put the consumers’ health in danger and cause perceptible side effects.

The study of the impact of adulterated honey on humans is noticeably important. However, human individuals cannot be used as a test model due to significant costs and harm toward human beings, and it is not ethically justified. Moreover, the common animal models, such as mice, rabbits, primates, and chickens, require plenty of time, money, and training of the operators. Therefore, in this study, the zebrafish (*Danio rerio*) has been selected as an animal test model due to high transparency, early life stage development, and cost reduction. 70% of genes which are associated with diseases in human body have functional homology in zebrafish [9]. While the early life stage of zebrafish (embryonic stage) can provide primary information, acute and chronic tests on adult zebrafish allow the observation of any sign regarding toxicity.

Based on previous research, carbohydrates are used in fish diets primarily as energy sources and for their binding properties. Starches, pectin, and hemicelluloses have pellet-binding characteristics of great importance to feed manufacturers. Therefore, carbohydrates may be added to the feed-in excess of the amounts that can be efficiently utilized for energy by the fish. As zebrafish are warm water omnivores, carbohydrates are likely an important component of their diet. Indeed, recent work on adult zebrafish has demonstrated that the amount of carbohydrate in the diet is positively correlated with growth rate. Eames, et al. [10] showed that zebrafish, similar to other omnivores, metabolize glucose faster than carnivorous teleosts. The relatively fast glucose metabolism of zebrafish should facilitate laboratory studies of pancreas and liver function. This literature proves that “carbohydrates” are the typical food component in the fish’s diet.

Thanks to the development of analytical methods, many studies have been conducted to detect food authentication and fraud. Here is a brief example of these studies; high-performance anion-exchange chromatography with pulsed amperometric detection (HPAEC-PAD) for the detection of CS, and stable carbon isotope ratio analysis (SCIRA) for the detection of high fructose syrup (HFS) [11], GC-MS for the detection of CS [12], cavity ring-down spectroscopy (CRDS) and isotope ratio mass spectrometry (IRMS) for the detection of CS [13], Raman spectroscopy for the detection of CS [14], matrix-assisted laser desorption/ionization mass spectrometry (MALDI-MS) for the detection of IS and CS [15], and headspace-gas chromatography coupled to ion mobility spectrometry (HS-GC-IMS) for the detection of CS [16]. On the other hand, ultrahigh-performance liquid chromatography coupled with quadrupole time-of-flight mass spectrometry (UHPLC/Q-TOF-MS) was applied for indirect adulteration of honey through bee feeding with multi-class sugar syrups, such as CS and IS [17]. Hence, the liquid chromatography-electrochemical detection (LC-ECD) [18] and laser-induced breakdown spectroscopy (LIBS) [19] methods were used for the detection of acacia honey blended with rape honey.

In this study, the LC_50_ and LD_50_ of *Heterotrigona itama* honey (acacia honey) and its sugar adulterants have been assessed and discussed, while histology provided depth observation through organs. In addition, the LCMS/MS-based metabolite shifting in the serum of adult zebrafish after treatment with pure and adulterated *H. itama* have been identified.

## 2. Results

### 2.1. Brix Value (%) Analysis

The Brix value (%) characterization of pure honey and sugar adulterated *H. itama* honey are presented in Table 1. The Brix value of pure *H. itama* honey was 65.40 ± 0.10, which was not significantly different (*p* > 0.05) from sugar adulterated *H.itama* honey with different concentrations (1, 2, and 3%).

### 2.2. Lethal Concentration (LC_50_) of Pure and Adulterated Honey

LC_50_ of pure and sugar-adulterated *H. itama* honey on zebrafish embryos at 96 hpf is shown in Table 2. Pure *H. itama* honey was adulterated with four types of sugar (cane sugar, inverted sugar, palm sugar, and light corn syrup in the concentration of 1%, 2%, and 3%, respectively). The LC_50_ of pure *H. itama* honey was 34.40 ± 1.84 (mg/mL), which was significantly (*p* ≤ 0.05) higher than sugar adulterated *H. itama* honey. Inverted sugar showed the lowest LC50 in all three concentrations among the whole sugar adulterants significantly (*p* ≤ 0.05), which indicated the highest toxic effect. The LC_50_ was decreased significantly (*p* ≤ 0.05) with increasing adulterant concentration. The inverted sugar at a concentration of 3% had the lowest LC_50_ (5.03 ± 0.92), followed by light corn syrup (7.60 ± 0.45), palm sugar (8.87 ± 1.90), and finally, cane sugar (9.87 ± 0.09) (*p* ≤ 0.05) (Table 2). Since there is no mortality observed in the control group (Embryo medium, E3), the result is not included in Table 2.

### 2.3. Mortality Rate

**Figure 1:**
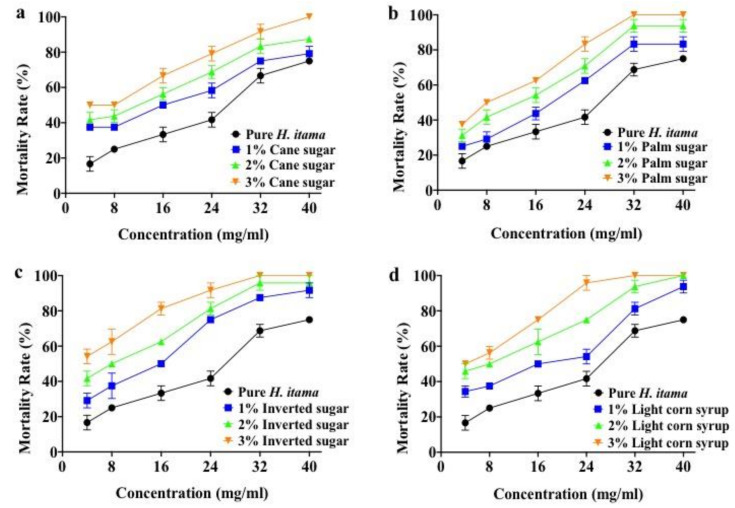
Dose-dependent effect of pure and sugar adulterated *H. itama* on zebrafish embryo mortality rates at 96 hpf: (**a**) cane sugar, (**b**) palm sugar, (**c**) inverted sugar, and (**d**) light corn syrup.

To evaluate the possible toxicity of adulterated honey on zebrafish embryos, the mortality rate as a developmental phenotype was analyzed. The mortality rate of zebrafish embryos after exposure to sugar adulterated *H. itama* honey at 96 hpf, is illustrated in Figure 1. Lethality was observed right after exposure in a dose-dependent manner between 4 and 40 mg/mL of pure and adulterated honey. The highest concentration (40 mg/mL) of sugar adulterated *H. itama* honey indicated 100% lethality of embryos in all four sugar adulterants at a concentration of 3%, which was significantly (*p* ≤ 0.05) different from pure *H. itama* honey.

### 2.4. Hatchability of Zebrafish Embryos

**Figure 2:**
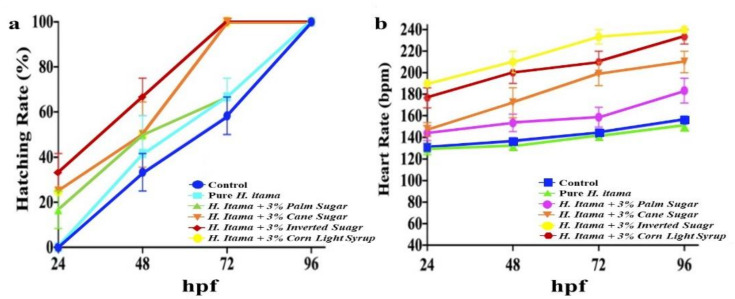
Concentration and time-dependent effect of pure *H. itama* honey on zebrafish embryos: (**a**) hatching rates and (**b**) heartbeat.

In general, hatching of the embryo into larvae happens from 48 to 72 hpf. The hatching rate (%) of pure and 3% sugar adulterated for *H. itama* honey is displayed in Figure 2a. The presented data shows that the early hatching rate of embryos increased significantly (*p* ≤ 0.05) in a time-dependent manner. Our results showed that the hatching rate of the sugar adulterated honey treated group started before 48 hpf, which is detrimental for fish.

### 2.5. Heartbeat Rate

The heartbeat rate of zebrafish embryos and hatched larvae measured at 24, 48, 72, and 96 hpf and statistical analyses are presented in Figure 2b. According to Figure 2b, there is a significant (*p* ≤ 0.05) increase in the number of heartbeats per minute (bpm) across embryonic development (24–96 hpf) treated with sugar adulterated *H. itama* honey in comparison to those treated with pure *H. itama* honey.

### 2.6. Acute Toxicity Test

**Figure 3:**
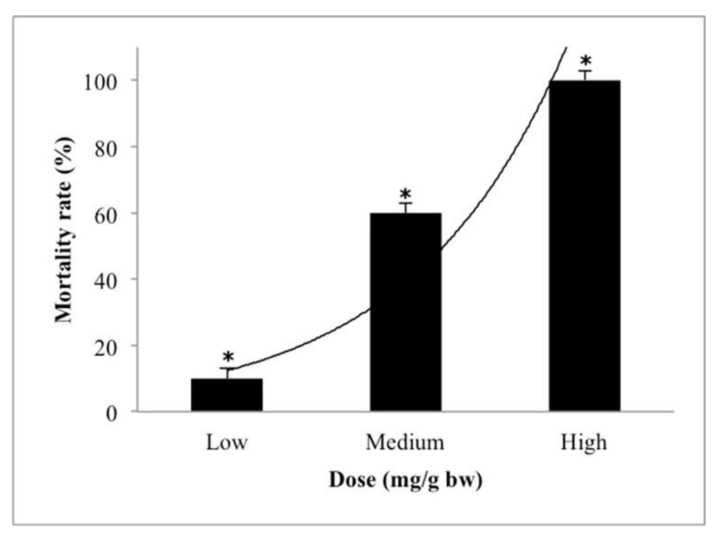
Dose-dependent effect of pure *H. itama* on mortality rate (%) of adult zebrafish during 24 h acute toxicity test. Low: 1 mg/g·bw, medium: 5 mg/g·bw, high: 10 mg/g·bw. Values significantly different are indicated by asterisks (* *p* ≤ 0.05).

The acute toxicity of pure *H. itama* honey in adult zebrafish (during the period of 24 h) in 3 different doses of low (1 mg/gbw), medium (5 mg/gbw), and high (10 mg/gbw) is presented in Table 3. The result in Table 3 showed that the safe dose for pure honey was at the low dosage with a mortality rate (%) of 10% for pure *H. itama* honey; Meanwhile, in the high dosage, 100% mortality rate (%) was reached for pure *H. itama* honey. However, the LD_50_ value of *H. itama* was 4.70 ± 1.77 (mg/g bw). Among the control group, no mortality was observed during the acute toxicity test. Figure 3 indicates the percentage of the mortality rate of pure *H. itama* during the acute toxicity test (24 h). The LD_50_ value was obtained by converting the percentage of mortality to probits (probability unit) and taking the logarithm of the concentrations in order to plot the probits versus the logarithm of the concentrations graph. Finally, by fitting the regression line, the LD_50_ value has been revealed.

### 2.7. Prolong-Acute Toxicity Test

**Figure 4:**
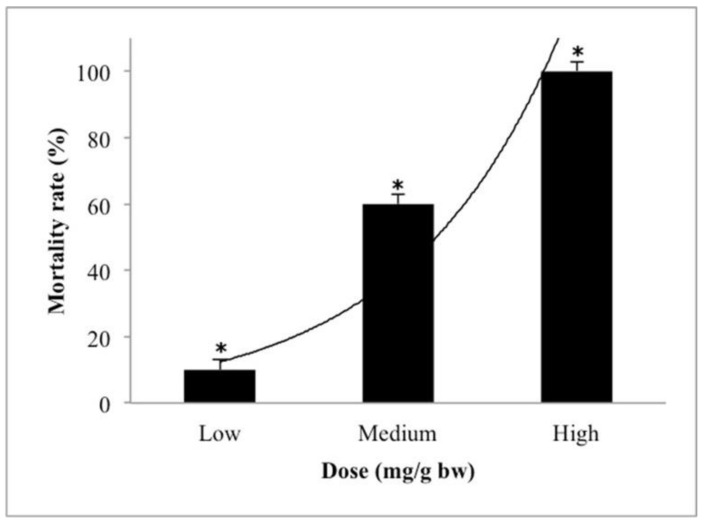
Dose-dependent effect of pure *H. itama* honey on mortality rate (%) of adult zebrafish during 72 h of the prolong-acute toxicity test. Low: 0.5 mg/g·bw, medium: 1 mg/g·bw, high: 3 mg/g·bw. Values significantly different are indicated by asterisks (* *p* ≤ 0.05).

Table 4 presents the prolong-acute toxicity of pure *H. itama* honey in adult zebrafish for 72 h in three different dosages of low (0.5 mg/g·bw), medium (1 mg/g·bw), and high (3 mg/gbw). The LD_50_ of pure *H. itama* honey was 5.22 ± 1.31 (mg/g·bw). Among the control group, no mortality was observed during the prolong-acute toxicity test. Figure 4 showed the mortality rate (%) of pure *H. itama* honey during 72 h of the prolong-acute toxicity test. There was a concentration-dependent response, and increasing the dosage of honey led to a rise in the mortality rate. Therefore, 0.5 and 1 (mg/gbw) dosages were chosen as the safe dose for the sub-acute study because less than 50% of zebrafishes were killed in this dose.

### 2.8. Sub-Acute Toxicity Test

Table 5 shows the median lethal dose (LD_50_) of pure and sugar adulterated *H. itama*. According to Table 5, inverted sugar with LD_50_ of 0.20 ± 0.06 (mg/mL) had the significantly (*p* ≤ 0.05) highest toxicity among all sugar adulterants, followed by light corn syrup (0.35 ± 0.07 mg/mL), palm sugar (0.80 ± 0.16 mg/mL), and cane sugar (1.13 ± 0.52 mg/mL). With the aim to determine the no observed adverse effect level (NOAEL), there was no lethality observed at a low dose for pure *H. Itama* honey after 14 days of the sub-acute toxicity test.

## 3. Histology

**Figure 5:**
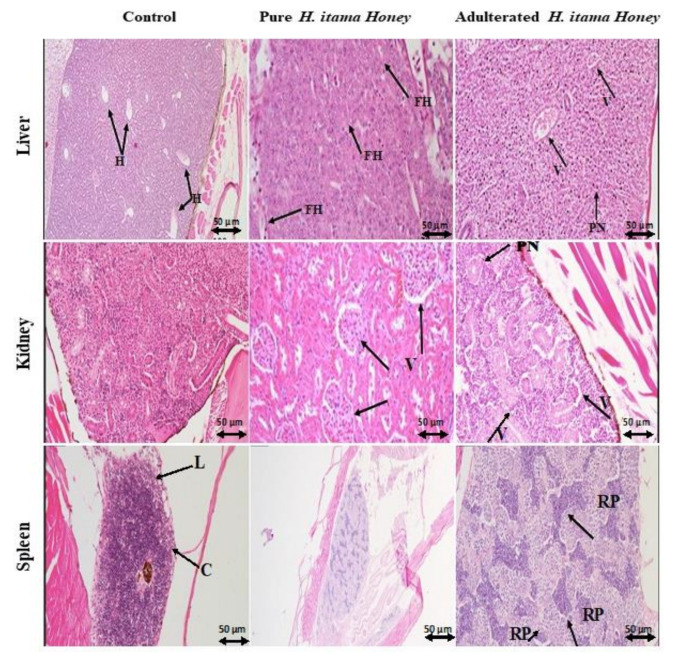
Histological alteration in the liver, kidney, and spleen of zebrafish treated with a different type of honey. Control groups with normal histological structure, the pure honey group with normal to the mild lesion, and the adulterated honey group with moderate to the severe lesion, H: hepatocyte, FH: foamy hepatocyte, V: vacuolation, PN: pyknotic nucleus, C: capsule, L: lymphoid tissue, RP: red pulp. H&E, bar = 50 μm.

Microscopic examination of the H&E-stained liver, kidneys, and spleen tissue of non-treated and treated zebrafish is shown in Figure 5. The liver of control and pure honey treated zebrafishes showed the normal structure of the hepatic lobules, hepatocytes, and sinusoids. In contrast, adulterated honey treated zebrafish groups exhibited several histopathological changes, including foamy hepatocytes, focal necrosis, and infarction, as well as a significant increase in lesion score. The kidneys of the control group showed fairly normal vacuolated renal tubules, while the kidneys of zebrafish from the adulterated honey group showed significant degenerative changes. The spleen tissue of the control group showed a connective tissue framework (which includes capsule, trabeculae, and reticular connective tissue) and parenchyma of lymphoid tissue in the form of white (lymphoid nodules) and red pulp and no significant lesion. The pure honey showed no significant lesion in the spleen, while adulterated groups showed depleted lymphocytes leading to small follicles and relatively large red pulp.

### 3.1. LC-MS Based Fingerprinting of Zebrafish Serum

**Figure 6:**
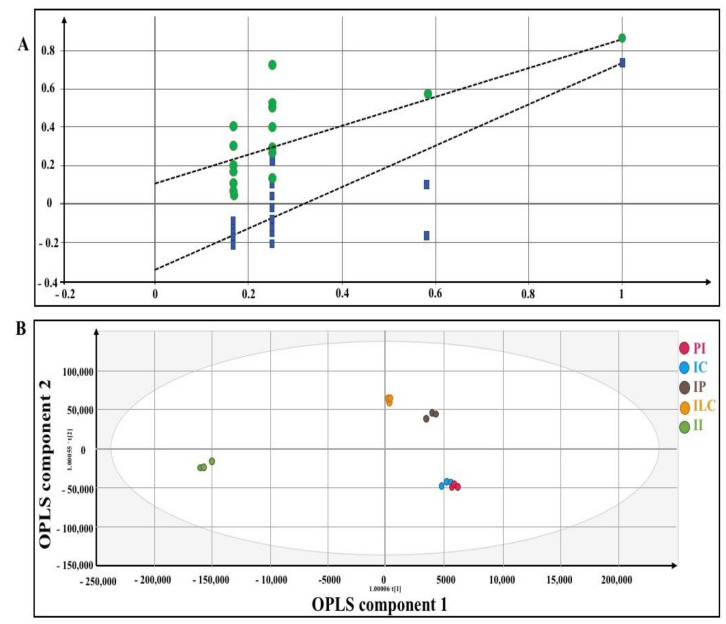
(**A**) Permutation results of partial least square-discriminant analysis (PLS-DA), (**B**) The OPLS score scatter plot of pure and adulterated *H. itama* honey using negative mode ionization in LC-MS analysis. PI = pure *H. itama* honey; IC = *H. itama* honey adulterated with cane sugar; IP = *H. itama* honey adulterated with palm sugar; ILC = *H. itama* honey adulterated with light corn syrup, and II = *H. itama* honey adulterated with inverted sugar.

The collected serum from different groups of zebrafish (pure and adulterated honey-treated groups) was analyzed using LC-MS to investigate their serum fingerprints. Multivariate data analysis with OPLS was performed on the pre-processed LC-MS dataset to observe the discrimination among the samples. Validation of the multivariate calibration was performed using permutation testing. Figure 6A shows the permutated model parameters and the R^2^ and Q^2^ values of the original model. The X-axis indicates the correlation coefficients between original and permutated models and Y-axis indicates the value of R^2^ and Q^2^. The original model exhibited higher values than those of the permutated models in the validation test. The X-axis denotes the correlation between original and permuted data response, whereas the Y-axis shows the R^2^ (the goodness-of-fit) and Q^2^ (predictability of the model). The permutation model is valid if the intercepts of R^2^ and Q^2^ are less than 0.4 and −0.05, respectively [20]. The R^2^ and Q^2^ values in this study were 0.114 and −0.342, respectively, which were in the acceptable range as shown in Figure 6A.

Figure 6B shows the score scatter plot displaying the discrimination of the pure *H. itama* honey and sugar adulterated *H. itama* honey-treated group. All of the pure and sugar adulterated honey-treated group were separated alongside OPLS components 1 and 2. While pure *H. itama* honey and cane sugar adulterated *H. itama* honey-treated group were shifted closer to each other in both OPLS-component 1 and 2 and support the LD_50_ results in which the cane sugar adulterated *H. itama* honey-treated group showed the lowest mortality rate after the pure *H. itama* honey-treated group. Inverted sugar adulterated-treated groups were shifted far from another honey-treated group alongside OPLS 1. This result is also in agreement with the result of a mortality rate that inverted sugar adulterated-treated group showed significantly (*p* ≤ 0.05) the lowest LD_50_ among other sugar adulterated-treated groups.

### 3.2. Structural Elucidation Using MS/MS Fragmentation

Metabolic profiling of zebrafish’s serum force-feed by pure and adulterated *H. itama* honey was analyzed by multivariate statistical analysis. Data were obtained by UHPLC negative ion in order to establish and validate metabolomic profiling models. The MS-MS fragmentation was used to elucidate the chemical structure of each parent ion, in combination with the search for the metabolites in HMDB, ChemSpider, and mzcloud. All fragmented MS spectra of each ion were compared to the NIST14 database library. By comparing all mass spectra, three biomarkers in negative ion modes were identified in Table 6.

## 4. Discussion

In this study, the screening procedure was optimized and standardized in order to determine the toxic concentration of specific adulterated *H. itama* honey using the zebrafish—embryo and adult—model. The embryonic stages of zebrafish have provided several scorable endpoints in a toxicological model, especially in mortality and hatching rate [21]. The results of this research prove that the type and concentration of adulterants could have a significant impact on the mortality rate (%) of the zebrafish embryo. The maximum LC_50_ was determined in order to monitor embryotoxicity at 96 hpf. The LC_50_ value for the toxicity of honey is indicated by the statistical estimation of the number of toxic substances (mg) per body weight (kg), which is required to be lethal to 50% of the population of the tested zebrafish embryos. According to the definition of LC_50_; the lower the LC_50_, the higher the mortality rate. Therefore, according to the results in Table 2, pure *H. itama* honey has higher LC_50_ and has significantly (*p* ≤ 0.05) less toxicity. Additionally, cane sugar is a less toxic sugar adulterant among other sugar adulterants, followed by palm sugar and light corn syrup. Inverted sugar in all adulterated percentages had the significantly (*p* ≤ 0.05) lowest LC_50_ value, which means higher toxicity compared to other sugar adulterants used in this paper. Soares, Amaral, Oliveira and Mafra [8] mentioned that adulterated honey with a high concentration of inverted syrup could cause harmful health effects, especially diabetes. Adulterated honey may lead to increased blood sugar, followed by the release of insulin hormone, causing insulin resistance and type II diabetes, abdominal weight gain and obesity, as well as a rise in the level of blood lipid and high blood pressure [22].

The development of zebrafish is categorized into four major stages: embryo, larvae, juvenile, and adult [23]. By 24 hpf, the embryos had functional circulatory systems, performed spontaneous twitching movements, and started pigmentation. Hence, the mortality rate investigation of zebrafish embryos began at 24 hpf. According to OECD (2012), the total percentage of the mortality rate was concluded by dead and unfertilized embryos, which revealed coagulated embryos, lack of somite formation, lack of heartbeat, and non-detachment of the tail. According to Figure 1, the mortality rate of embryos reached the maximum level at 96 hpf in sugar adulterated honey groups. Adenan, et al. [24] stated that a higher concentration of *H. itama* honey could cause a higher incidence of lethality and abnormality at the early life stage of zebrafish embryos due to coagulation and absence of heartbeat in the zebrafish embryo. With regard to Figure 1, the mortality rate was recorded from 24 hpf onward, and by increasing the concentration of pure *H. itama* honey from 4 mg/mL to 40 mg/mL, a significant rise was experienced in the mortality rate of embryos (*p* ≤ 0.05). Exposure to 40 mg/mL of adulterated honey induced a significant (*p* ≤ 0.05) increase in mortality rate among different percentages (1, 2, and 3%) of sugar adulterated honey (Figure 1). These results clearly showed that the number of dead embryos was correlated to the type of adulterants and the duration of treatment (hpf). In the liver of zebrafish, α and β cells, which are responsible for increasing glycogenesis, are generated at 24 hpf and matured at the same as in mammals [25]. Glycogenesis elevates the glucose level in embryos, which may cause an increase in the mortality rate in sugar adulterated *H. itama* honey, as is shown in Figure 1. In over-nutrition diets, such as high glucose concentration, the number of β cells is boosted in both embryos and juvenile zebrafish [25]. In general, glucose may cause reactive oxygen species (ROS) production in the body through various ways in mitochondria, such as nicotinamide adenine dinucleotide phosphate (NADPH)-oxidase, sorbitol pathway, activated glycation, and insulin pathway [26]. High-glucose concentration triggered NADPH-oxidase [27] and caused a reduction of O_2_ to O_2_^−^ and H_2_O_2_ to OH [26], as shown in Equation (1):

(1) $$2O_{2}+\mathrm{NADPH}\overset{\mathrm{NADPH}-\mathrm{Oxidase}}{\to }\;2O_{2}^{-}+{\;\mathrm{NADP}}^{+}+{\;H}^{+}$$

On the other hand, insulin may be secreted in response to the consumption of sugar-adulterated honey. It would activate NADPH-oxidase located in the membrane enzyme system. Additionally, sugar consumption generates ROS in the body, which is a toxic element and can cause chronic diseases such as atherosclerosis, cancer, diabetes, hypertension, coronary artery diseases, and heart failure. Therefore, this may explain the high mortality rate of zebrafish embryos treated with sugar adulterated *H. itama* honey when adulteration percentages were increased, as seen in Figure 1. In addition, Jurczyk, et al. [28] mentioned that glucose level reached the highest peak in the embryo at 96 hpf due to glucagon production, which stimulated gluconeogenesis and was expressed in enteroendocrine cells of the gut. This could be a reason the mortality rate (%) of embryos treated with sugar adulterated *H. itama* honey rose at 96 hpf, as seen in Figure 1. The naturally produced glucose inside the body or adding glucose to the food can be harmful to zebrafish. These findings indicated that glucose toxicity in the zebrafish model may be used to suggest pathways with relevance to human diseases, especially diabetes.

Hatching is a critical point in a fish’s life cycle that illustrates the developmental toxicology and biochemical mechanisms [29]. According to Figure 2a, control and pure *H. itama* honey followed the same hatching rate trend at 24–48 hpf. While zebrafish embryos treated with sugar adulterated *H. itama* honey demonstrated a high early hatching rate at 24 hpf. It should be considered that early hatching at 24 hpf was detrimental for zebrafish embryos since non-fully-developed larvae cannot survive exposure to the external environment. The mortality of individuals exposed to a high dose of adulterated honey may be explained by a high glucose load. These findings are concurrent to previous studies on rats that long-term consumption of any sugar syrup, such as sucrose or fructose, demonstrated a high mean total percentage of body fat and increased visceral fat pads, which led to hypercholesterolemia, hypertriglyceridemia, and hyperinsulinemia and caused animal death [30]. Therefore, these may be a reason for the high mortality rate of zebrafish by sugar adulterated *H. itama* honey when the dosage of adulterants was increased (Figure 2a). Furthermore, simple sugars, such as glucose and fructose—which naturally exist in pure honey—supply instant energy to human body cells and have a low glycemic index compared to sucrose as a complex sugar [31]. Pure honey showed significantly (*p* **≤** 0.05) lower toxicity in Figure 2a due to containing not only simple sugar (glucose and fructose) but also other nutrients, such as proteins, antioxidants, and minerals, which are essential to human health [32]. Zebrafish is a unique and vital model for the genetics and drug-driven heart failure due to the similarity of its heart to the human heart (Dahme, Katus, and Rottbauer, 2009). Therefore, in this paper, we developed and described the effect of pure and adulterated honey on the cardiac rate of zebrafish embryos. The regular heartbeat rate of zebrafish embryos varies between 120–180 bpm. A lower or higher heartbeat rate will cause embryo death. As has been demonstrated in Figure 2b, there was a slight decrease in the cardiac rate for pure *H. itama* honey compared to the control group that was due to the antioxidant activity of honey. Hence, honey, as a source of various antioxidant parameters, can reduce heartbeats to the normal level (60–90 bpm), which is beneficial not only for patients suffering from tachycardia (heart rate > 100 bpm) but also hypertension and diabetes mellitus patients. There was a significant (*p* ≤ 0.05) increase in zebrafish embryo cardiac rate above the normal level at 96 hpf (>180 bpm) when treated with sugar adulterated *H. itama* honey (Figure 2b). The zebrafish embryo treated with inverted sugar adulterated *H. itama* honey indicated the highest heartbeat rate compared to other sugar adulterated *H. itama* honey treated groups (Figure 2b). The rise in the heartbeat of embryos could be due to the presence of cardiac glycoside compound in sugar adulterated *H. itama* honey. According to Prassas and Diamandis [33], cardiac glycoside causes the inhibition of Na^+^/K^+^-ATPase and leads to raising the level of calcium ion to elevate the cardiac contractile force.

A model of force-feeding of adult zebrafish was appointed successfully to investigate the impact of pure and adulterated honey on the mortality rate (%). Pure honey significantly (*p* ≤ 0.05) showed less toxic impact, which might be due to the existence of simpler sugars and high levels of antioxidants compared to adulterated honey (Table 5). The mortality of individuals exposed to a high dose of adulterated honey may be explained by a high glucose load. The LD_50_ findings in Table 5 were in agreement with our embryo toxicity test (LC_50_) presented in Table 2. Hence, glucose is either naturally produced in the body or added to foodstuff and can be detrimental to zebrafish. These findings persuaded us that glucose toxicity in the zebrafish model may identify pathways with relevance to human diseases, especially diabetes. Turanose, 3-O-α-D-glucosyl-D-fructose is one of the sucrose isomers that naturally exists in honey. The 14-day acute and 13-week subchronic oral toxicological study of turanose showed no toxic effects on mice, and the LD_50_ value was greater than 10 g/kg b.w (Chung 2017). The duration of honey consumption and the dosage may play a major role in the outcome of current results.

Histopathological examination is still considered the major standard in order to assess fibrosis and lesion occurrences in internal organs, such as the liver, kidney, spleen, etc. Typical histopathological alteration of sub-acute toxicity (14 days) is presented in Figure 5 individually for all non-treated and treated groups exposed to pure and adulterated honey. The liver is the first organ susceptible to poison and toxic substances. Any abnormality in the appearance of the liver is diet-dependent. In detail, the pure honey treated group showed mild foamy hepatocytes, while the remaining adulterated honey groups had vacuolation of the hepatocytes, affecting almost all hepatocytes. The adulterated honey groups also showed individual pyknotic nuclei scattered throughout the liver section. The liver of zebrafish treated with adulterated honey had huge hematomas. The hepatocytes of all control zebrafish appeared normal. The liver sections of all honey-treated (pure and adulterated) groups showed some degree of blood vessel congestion. The pure honey groups had relatively mild congestion, while the adulterated groups showed severe congestion. The long-term (14 days) exposure to adulterated honey led to the prolonged generation of reactive oxygen species (ROS), which caused hepatic injury, and confirmed a higher mortality rate in the adulterants treated group (Figure 5). Once a poison is ingested, it is absorbed from the intestine and is brought to the liver for detoxification. This results in congestion in the liver and also changes in liver architecture, followed by loss of tissue function as a response to ROS. On the contrary, there was no alteration in liver tissue of the control group (Figure 5). Ranneh, et al. [34] indicated a reduction of inflammatory cell infiltration in the liver of Sprague–Dawley rats when supplemented with stingless bee honey (*Trigona*). However, in current findings, only foamy hepatocytes and pyknotic nuclei were present in the liver of zebrafish fed with pure honey (Figure 5). Such conflicting results might depend on the type of toxic compound and its concentration, animal species, exposure time, etc. [35]. Among these pathological changes, hepatocytes initially showed vacuolation then necrosis in the form of the pyknotic nucleus, which was indicated in Figure 5. In this regard, Ni, et al. [36] suggested vacuole formation in the hepatocyte might be due to interference in lipid metabolism followed by fat filtration in the liver of zebrafish, while hepatocyte necrosis and apoptosis might cause the pyknotic nucleus. From the liver, the poison circulates throughout the body. However, the poison is eventually removed from the body through the kidney. Hepatocyte lesion reduces liver efficacy to detoxify the poison leading to congestion and degenerative changes in the kidneys, as observed in Figure 5. Lesions in the kidneys were observed mostly in the tubules, the epithelial lining, and the interstitial tissue, particularly the blood vessels. The tubular epithelium showed various degenerative changes, including cytoplasmic vacuolation, cytoplasmic destruction, and pyknotic nuclei. The blood vessels showed various degrees of congestion as a result of hypercellular activities in the kidney head. Regarding the tubular vacuolation (mild to moderate) in the adulterated-honey treated group, Kiss and Hamar [37] emphasized tubular vacuolization as a sign of toxicity. However, experimental studies support sugar intake as one of the major factors causing kidney injury. With regard to this, Johnson, et al. [38] found the administration of a 60% fructose diet to rats induces renal hypertrophy with tubular cell proliferation and low-grade tubulointerstitial injury. Osmotically active compounds, such as some sugars, often result in “osmotic nephrosis”, which appears as cytoplasmic vacuolation of renal tubule epithelium and risk factors for the development of pre-existing chronic kidney diseases (CKD).

The large red pulp appearing in the spleen of the adulterated treated group (Figure 5) could be due to congestion of blood cells by toxic compounds from adulterants. In this regard, Bronte and Pittet [39] mentioned that congestion in the spleen leads to blood accumulation in the red pulp, which, consequently, appears to be much more extensive than when the organ is first emptied of its blood, leading to the death of the fish.

The three metabolites were identified through LC-MS/MS analysis from the serum of zebrafish, as shown in Table 6, which were force-fed with pure and sugar adulterated *H. itama* honey. The identified metabolites were: (1) S-Cysteinosuccinic acid from pure *H. itama* group, (2) 2,3-Diphosphoglyceric acid from adulterated *H. itama* with cane sugar, and (3) Cysteinyl-Tyrosine from adulterated *H. itama* with palm sugar. These identified metabolites were counted as endogenous biomarkers while consuming pure or adulterated honey. S-Cysteinosuccinic acid was detected in the pure *H. itama* honey-treated group (Table 6). This endogenous compound was also observed at low levels in the urine of Sprague–Dawley rats with diet-induced hyperlipidemia (high-fat diet) compared with the control group [40]. In another study, Chakrabarti and Denniel [41] reported the toxicity of S-conjugated Cysteine (*S*-[(1 and 2)-phenyl-2-hydroxyethyl]-cysteine) in the rat. They observed a sequence of systematic toxic responses in renal proximal tubules of the rat. In addition, this research suggested that stimulation of lipid peroxidation mitochondria due to the S-conjugated Cysteine produced malondialdehyde (MDA) exposure in renal proximal tubules would lead to cell death. Therefore, it can be concluded that the S-conjugated Cysteine compound can alter the mitochondrial function as a result of lipid peroxidation. This function alteration leads to cell death due to the consequent loss of cellular energy supplies as a result of LD_50_ (Table 5). Furthermore, the fairly vacuolated accrued renal tubules of the pure *H. itama* honey-treated group in Figure 5 could be due to the accumulation of this metabolite in zebrafish. 2,3-Diphosphoglyceric acid, known as 2,3-DPG, was detected from cane sugar adulterated *H. itama* honey-treated groups (Table 6). This biomarker is present in human blood at high levels and acts as an allosteric effector in order to release more oxygen from haemoglobin and leads oxygenation to the tissue [42]. Macdonald [43] proposed that 2,3-DPG accumulation might be due to hyperthyroidism and an increased rate of glycolysis. Additionally, a high accumulation of 2,3-DPG has been reported in patients with congestive cardiac failure, chronic renal failure, and cirrhosis of the liver [43]. Since this compound has been identified in treated zebrafishes with cane sugar adulterated *H. itama* honey, the liver failure as a result of huge hematomas, significant degenerative changes in the kidney (Figure 5), and lower LD_50_ values (Table 5) in comparison to pure *H. itama* could be explained. Furthermore, Cysteinyl-Tyrosine was detected in the serum of zebrafishes that were treated with *H. itama* honey adulterated with palm sugar (Table 6). Cysteinyl-tyrosine covalent could be found in cysteine deoxygenase (CDO), galactose oxidase, and NirA [44,45,46]. The Cys and Tyr concentration alteration in blood serum is associated with insulin resistance. Therefore, it is a noticeable biomarker in asymptomatic syndromes, such as adiposity, hyperglycemia, hypertension, and dyslipidemia [47]. These symptoms can be related to pathological changes in the liver of zebrafish treated with adulterated *H. itama* honey, as shown in Figure 5.

However, the current study was the first in vivo toxicity study of pure and adulterated *H. itama* honey on zebrafish—embryo and adult; thus, there is a lack of established protocol in the zebrafish model, causing an inability to make significant comparisons among various studies.

## 5. Materials and Methods

### 5.1. Materials

The cane sugar, palm sugar, and light corn syrup were attained from Malayan Sugar Manufacturing Prai Bhd (Sungai Buloh, Selangor, Malaysia). Nek Nor and Phoon Huat & Co. Pte. Ltd. (AEON Supermarket, Kuala Lumpur, Malaysia), respectively. With regard to force-feed apparatuses, a 22-G soft tube catheter was purchased from SAI Infusion Technologies (Chicago, USA) and a 1 mL luerlok syringe was procured from Hamilton (Reno, Nevada). Formalin, ethanol, and glacial citric acid were procured from Sigma Chemical Co. (St. Louis, MO, USA). Absolute methanol and formic acid (HPLC grade) were purchased from Thermo Fisher Scientific (Pittsburg, PA, USA). Distilled water was passed through a Milli-Q water purification system (Millipore, Bedford, MA, USA) for the preparation of LC mobile phases.

### 5.2. Methods

#### 5.2.1. Sample Collection

The pure honey sample was collected fresh from a local honey seller located at Gong Beris, Terengganu, Malaysia from an acacia environment—Acacias. The honey sample was kept at 4 °C during the transportation and upon arrival at the lab. The sampling method of *H. itama* honey was adopted from the Department of Standard Malaysia (Malaysian Standard, 2017).

#### 5.2.2. Brix Analysis

Total soluble solid was measured at 25 °C using a digital refractometer (ATAGO, Tokyo, Japan). Approximately 0.3 mL of honey was placed onto the prism surface, and the reading was recorded as Brix [48].

#### 5.2.3. Honey Adulteration

Pure honey was filtered by 60 mesh screens in order to eliminate impurities and was stored in the dark at 4 °C for further analysis. Before the analysis, the honey sample was thermostated to room temperature (25 °C) and homogenized by manual stirring thoroughly for at least 3 min. Sugar adulterants (light corn sugar, cane sugar, inverted sugar, and palm sugar) each at 1%, 2%, and 3% (*w/w*) of total volume were added to the pure *H. itama* honey.

#### 5.2.4. Embryotoxicity Study in Danio Rerio

The zebrafish embryotoxicity assay was carried out based on the Organization for Economic Cooperation and Development (OECD) guideline for fish embryo toxicity (da Silva, Gauche, Gonzaga, Costa, and Fett) test (OECD, 2012). Wild-type zebrafish embryos were procured from Danio Assay Laboratories Sdn.Bhd (Serdang, Selangor, Malaysia).

#### 5.2.5. Toxicity Test

Different concentrations (4, 8, 16, 24, 32, and 40 mg/mL) of adulterated honey and pure honey (as control) were set in 96-well plates (Thermo Scientific TM, New Hamshire, UK). Fertilized zebrafish eggs were collected within 3 h of postfertilization (hpf). The embryo was pipetted into each well by using a micropipette (Eppendorf, Hamburg, Germany), i.e., four replications (*n* = 8). Embryo medium E3 was used as a positive control. The medium was not renewed throughout the experiment. Embryo development was observed at 24, 48, 72, and 96 hpf with an inverted microscope (Nikon, Eclipse TS100, Chicago, IL, USA) to assess mortality. Mortality was scored by two complementary criteria, including the coagulation of embryos at early stages (24 and 48 hpf), and deformity sufficiently severe (e.g., lack of somite and lack of heartbeat) to be considered effectively lethal (OECD, 2012). The numbers of embryos with lethal effects were scored at 96 hpf in all test groups. The mortality rate (%) of embryos was determined using Equation (2) as below:

Mortality rate (%) = No. of dead embryos/Total embryos × 100(2)

#### 5.2.6. Evaluation of Zebrafish Embryos Hatching Rate

The zebrafish embryo hatch rate was observed at 24, 48, 72, and 96 hpf with an inverted microscope (Nikon, Eclipse TS100, Chicago, IL, USA). The hatching rate of embryos was determined as the rupture of the chorion for release of larvae in the comparison between different pure and adulterated honey (3%). The hatching rate (%) was calculated using Equation (3) as follow:

Percentage of hatchability (%) = No. of hatched embryos/total number of incubated embryos × 100(3)

#### 5.2.7. Evaluation of Zebrafish Embryo and Larvae Heartbeats

The heartbeat of zebrafish embryos and larvae at 24, 48, 72, and 96 hpf was examined after treatment with pure and adulterated honey (3%). The cardiac toxicity was assessed by manually counting each embryo and larvae heartbeat within 1 min by direct visual observation of the zebrafish embryo and larval cardiac ventricles by using an inverted microscope connected to a computer and camera device using objective lenses with 4×, 10×, and 40× magnification, and the heart rate was counted per minute.

#### 5.2.8. Pre-and Post-Treatment Zebrafish Maintenance

The aim of the adult zebrafish toxicity test is to find out the critical dose that kills 50% of zebrafishes (LD50). It would lead to discovering the safe dose in which the zebrafish will survive and guarantee a specific concentration of adulterated honey safe for human consumption.

The study was approved under the Ethical Use of Animals (IACUC) with Reference No. AUP-R059/2018. All tested fishes were selected from a laboratory population from a single stock. The fishes were acclimated for two weeks prior to the test under conditions of water quality and illumination similar to those of the test condition OECD [18]. Force-feeding or oral administration was obtained by Collymore, et al. [49] for this study with slight modification. Briefly, adult zebrafishes with an average weight of 0.5–0.8 g, an average length of 24.50 mm, and 4 months old were purchased from Danio Assay Laboratories Sdn. Bhd. (Serdang, Selangor, Malaysia). Zebrafishes were fed daily with fish micropellet and live food (Artemia) and maintained in aquaria at the temperature of 28°C with a 14:10 h light-dark cycle. Zebrafishes were fasted for 12 h prior to force-feeding [50] in order to evacuate the intestinal bulb. On the day of force-feeding, fifteen randomly selected zebrafish (Danio rerio) were force-fed with different concentrations of honey. It was suggested that at least seven fish must be used for each test concentration and control, therefore, a total of 15 fish were used in the present study. The selection of adulterant’s concentration was based on the result obtained from the toxicity test on zebrafish embryos. The LD_50_ value was expressed in terms of the weight of the test substance per unit weight of the test animal (mg/kg) [51] for all three types of toxicity tests (acute, prolong-acute, and sub-acute). The median lethal dose (LD_50_) is a statistically derived single dose of a substance that can be expected to cause death in 50% of animals when administered by the oral route.

#### 5.2.9. Design of The Acute Toxicity Test

An acute oral toxicity test was performed to observe the short-term administration of honey according to the guidelines of OECD for testing of chemicals, TG 423 with slight modifications [51]. It measures the toxic effects of honey, such as lethality, occurring from oral administration of a single dose of honey. For the test group, fixed doses of pure *H. itama* honey with a concentration of 1, 5, and 10 mg/g body weight (BW) of adult zebrafish were force-fed only once, and the control group was force-fed with distilled water. Meanwhile, mortality was observed after 24 h.

#### 5.2.10. Design of The Prolong-Acute Toxicity Test

The prolong-acute toxicity test of pure *H. itama* honey on the adult zebrafish was carried out for 3 days (72 h) according to Khedkar, et al. [52] with slight modification. Dosing for the prolong-acute procedure was reduced to 3, 1, and 0.5 mg/g BW of the zebrafish. The mortality rate (%) of zebrafishes was recorded at the end of 72 h.

#### 5.2.11. Design of The Sub-Acute Toxicity Test

To determine the long term effect of the honey toxicity on the survival rate of adult zebrafish, they were exposed to the sub-acute toxicity test with different concentrations of pure and adulterated honey solutions for 14 days according to Ni, Peng, Gao, Ji, Ma, Li and Jiang [36] with slight modification. Multi force-feeding was applied regarding a hypothesis by Olsen, et al. [53] that adult zebrafish would be capable of pancreatic regeneration. To prevent regeneration and allow the toxicity effect to occur, zebrafishes were force-fed at days 1, 5, 9, and 13 with a dose of 0.1, 0.5, and 1 mg/g BW of the zebrafish. Mortality rate (%) was recorded on day 14, and safe concentrations at which more zebrafishes survived were chosen as a safe dose.

## 6. Histology

Various organs (liver, kidney, and spleen) of zebrafish underwent fixation and embedding steps for observation process by a skilled pathologist who was blind to the study in order to evaluate the pathological changes and scored inflammation.

### 6.1. LC-MS-Q TOF Based Fingerprinting

On day 14 after the sub-acute toxicity test, the zebrafish were fasted for 24 h prior to blood withdrawal. While previous studies developed various protocols for blood collecting, such as lateral incision in the dorsal aorta region, decapitation, and tail ablation, which required the animal to be sacrificed [10], we proceeded with blood collection from the heart of live zebrafish through the gills. The blood was withdrawn from the heart using a syringe, the needle was pushed carefully under the gill, and around 10 µL of the blood was withdrawn. The zebrafish blood was centrifuged (Thermo ScientificTM, New Hamshire, UK) under 10,000 rpm for 10 min to separate the serum (supernatant) from the debris (precipitate). The serum was immersed in liquid nitrogen for enzyme inactivation. Subsequently, 5 µL of the serum was added into 250 µL of water: methanol (1:1, *v/v*), vortexed and centrifuged at 10,000 rpm for 10 min. Then, 200 µL of the supernatant was transferred into the LC-MS insert vial and stored at −80 °C prior to the LC-MS/MS analysis. The prepared samples were subjected to metabolite identification using an Agilent 1290 Infinity LC system coupled with Agilent 6530 Accurate-Mass Quadrupole-Time-of-Flight (QTOF) mass spectrometry (Agilent, Santa Clara, CA, USA). The serum sample (10 µL) was injected into the UPLC-Q-TOF-MS system. A C18 column (Phenomenex, Torrance, CA, USA) of 100 Å, and 250 × 4.6 mm, 5µ) was used as a stationary phase. The mobile phase was composed of solvent A (100% CH_3_OH) and solvent B (a mixture of H_2_O and 0.1% CH_2_O_2_). The run started initially at 95% of solvent A and 5% of solvent B for 1 min, then from 95% to 64% of solvent A and 5% to 36 % of solvent B in 2–16 min, followed by 0% of solvent A and 100% solvent B at 17–27 min. The final run was 95% of solvent A and 5% of solvent B in 28–33 min. The total run time used was 33 min. Electrospray ionization with negative mode without fragmentation was applied.

The data was pre-processed using ACD/Spec Manager v.12.00 lab software (Advanced Chemistry Development, Inc., Toronto, Canada) and converted into a CDF file. The mass net CDF files were further pre-processed using Rstudio (Boston, MA, USA) [54] to extract all the related information from the raw data and convert it to a data matrix, which includes systematic noise filtering, automatic peak detection, baseline correction, data binning, deconvolution, and chromatographic alignment. Finally, the data were summarized into an Excel sheet prior to multivariate data analysis [55].

### 6.2. Statistical Analysis

Following recommendations in the OECD guideline (OECD, 2012), probit analysis was used to determine LC_50_ and LD_50_. All statistical analyses were expressed as mean ± standard error of the mean using GraphPad Prism 8.0 for Macintosh (GraphPad Prism, La Jolla, CA, USA) to calculate LC_50_ and LD_50_ based on the concentration-response relationship. To distinguish the statistically significant difference between treatment and control groups, one-way ANOVA followed by Tukey’s test was performed. *p* ≤ 0.05 was considered significant. For chemometrics analysis, the pre-processed raw data were imported using SIMCA P + 14.0 software (version 14.0, Umetrics, Umea, Vasterbotten, Sweden) and a model was developed using the OPLS method. Subsequently, the model was validated through a permutation test. Finally, the score scatters were developed to identify potential endogenous metabolites. All experiments were conducted in triplicate.

## 7. Conclusions

According to a significant (*p* ≤ 0.05) increase in mortality rate (%) of zebrafish—embryo and adult—in sugar adulterated *H. itama* honey, our experiment proved that adulterated *H. itama* honey is not beneficial toward human health, while pure honey does not show any significant (*p* ≤ 0.05) cardiotoxicity effect. However, the novel information of this study revealed the dose-dependent effect of adulterated honey on the heartbeat and hatching rate of zebrafish embryos. The tissue modification of adult zebrafish may ring the bell for upcoming research on human tissue alteration due to the consumption of adulterated honey. The results of this study demonstrate that adulterated honey possesses some endogenous compounds that are harmful toward the zebrafish body. Considering all approaches, these results might be a promising candidate for early diagnostic biomarkers that can prevent the development of metabolic diseases, such as diabetes (type 1 and 2). The findings of this study can be extrapolated to human health, and it can be concluded that adulterated honey only enhances honey flavor and acceptability by consumers. Since this is the first research on the toxicity effect of sugar adulterated *H. itama* honey in an aquatic system, these findings can be used to provide some guidance regarding risk assessment and an acceptable safe level of adulterants. In order to gain valuable insight regarding the toxicity of adulterated honey on internal organs, a more in-depth study of the embryo and juvenile zebrafish is warranted for future studies.

## Figures and Tables

**Table 1:** The Brix value (%) of *H. itama* honey adulterated with different sugars at different concentrations.

Samples	(%)	Brix (%)
Pure *H. itama* honey	0	65.40 ± 0.10 ^abc^
Palm sugar	1	64.92 ± 2.10 ^abc^
	2	64.62 ± 1.49 ^bc^
	3	66.04 ± 0.19 ^ab^
Cane sugar	1	64.72 ± 1.12 ^bc^
	2	65.41 ± 1.16 ^abc^
	3	65.53 ± 0.49 ^abc^
Light corn syrup	1	66.14 ± 0.34 ^ab^
	2	66.46 ± 0.57 ^a^
	3	65.83 ± 0.95 ^ab^
Inverted sugar	1	64.08 ± 0.72 ^c^
	2	65.56 ± 0.60 ^abc^
	3	65.69 ± 0.44 ^ab^

Values within the same column with different letters (a–c) are significantly different (*p* < 0.05).

**Table 2:** The LC_50_ of pure and sugar adulterated *H. itama* honey at 96 hpf.

Sugar Adulterant	% Sugar	LC_50_(mg/mL)
Control	0	34.40 ± 1.84 ^a^
Cane Sugar	1	18.94 ± 1.17 ^b^
	2	12.51 ± 1.73 ^c^
	3	9.87 ± 0.09 ^d^
Palm Sugar	1	14.25 ± 1.17 ^e^
	2	11.98 ± 0.66 ^f^
	3	8.87 ± 1.90 ^g^
Light corn syrup	1	13.52 ± 0.71 ^h^
	2	10.12 ± 1.18 ^i^
	3	7.60 ± 0.45 ^j^
Inverted sugar	1	10.66 ± 0.40 ^k^
	2	8.29 ± 1.00 ^l^
	3	5.03 ± 0.92 ^m^

Note: Values within the same column with different letters (a–m) are significantly different (*p* < 0.05).

**Table 3:** Acute toxicity test of pure honey *H. itama* in adult zebrafish (*Danio rerio*).

Sample	Dose (mg/g·bw)	Total Fish Tested	Mortality (%)	LD_50_ (mg/g·bw)
*H. itama*	Low	15	10	4.70 ± 1.77
	Medium	15	60	
	High	15	100	
Fish tank water	-	15	0	-

Note: Data are shown as the mean ± SEM. Low: 1 mg/g·bw, medium: 5 mg/gbw, high: 10 mg/gbw. LD: lethal dose; hpf: hour post-fertilization; SEM: standard error of the mean.

**Table 4:** The prolong-acute toxicity test of pure *H. itama* honey in adult zebrafish (*Danio rerio*).

Sample	Dose (mg/g bw)	Total Fish Tested	Mortality (%)	LD_50_ (mg/g·bw)
*H. itama*	Low	15	10	5.22 ± 1.31
	Medium	15	30	
	High	15	100	
Fish tank water	-	15	0	-

Note: Data are shown as the mean ± SEM. Low: 0.5 mg/g·bw, medium: 1 mg/g·bw, high: 3 mg/g·bw. LD: lethal dose; hpf: hour post-fertilization; SEM: standard error of the mean.

**Table 5:** Sub-acute toxicity test of pure and sugar adulterated *H. itama* honey in adult zebrafish (*Danio rerio*) after 14 days.

Sample	Dose (mg/g BW)	Total Fish Tested	Mortality (%)	LD_50_ (mg/mL)
*H.itama*	Low	15	0	2.33 ± 0.24 ^a^
	Medium	15	20	
	High	15	40	
*H.itama* + 3%Cane sugar	Low	15	20	1.13 ± 0.12 ^b^
	Medium	15	40	
	High	15	50	
*H.itama* + 3%Palm sugar	Low	15	30	0.80 ± 0.16 ^c^
	Medium	15	30	
	High	15	60	
*H.itama* + 3%Light corn syrup	Low	15	30	0.35 ± 0.07 ^d^
	Medium	15	30	
	High	15	80	
*H.itama* + 3%Inverted sugar	Low	15	30	0.20 ± 0.06 ^e^
	Medium	15	60	
	High	15	100	
Fish tank water	-	15	0	-

Note: Data are shown as the mean ± SEM. Means with different small letters (a–e) in the same column are significantly different (*p* ≤ 0.05). Low: 0.1 mg/g·bw, medium: 0.5 mg/g·bw, high: 1 mg/g·bw. LD: lethal dose; hpf: hour post-fertilization; SEM: standard error of the mean.

**Table 6:** Tentative endogenous metabolites in the serum of zebrafish fed with pure and adulterated honey were identified using LC-MS/MS fragmentation based on negative mode ionization.

Compound	M-H	MSMS Fragment Ions	Tentative Metabolites
1	236.00	[M-CHO_2_]^−^ at *m/z* 192, [M-CH_3_O_2_]^−^ at *m/z* 190, [M-C_2_H_5_O_2_]^−^ at *m/z* 176	S-Cysteinosuccinic acid
2	264.95	[M-OH]^−^ at *m/z* 246, [M-CO_2_]^−^ at *m/z* 220, [M-H_2_]^−^ at *m/z* 218, [M-CH_3_O_2_]^−^ at *m/z* 216, [M- C_3_H_2_O_6_P]^−^ at *m/z* 98, [M-C_3_H_4_O_6_P]^−^ at *m/z* 96	2,3-Diphosphoglyceric acid
3	283.00	[M-H_2_O]^−^ at *m/z* 265, [M-CO_2_]^−^ at *m/z* 239, [M-CH_2_O_2_]^−^ at *m/z* 237, [M-H_4_OS]^−^ at *m/z* 231, [M-CH_3_O_2_]^−^ at *m/z* 246	Cysteinyl-Tyrosine

## Data Availability

The data presented in this study are available on request from the corresponding author. The data are not publicly available due to the rules and regulations of University Putra Malaysia (UPM).

## Sample Availability

Samples of the compounds are available from the authors.
